# Distinct network topology in Alzheimer’s disease and behavioral variant frontotemporal dementia

**DOI:** 10.1186/s13195-020-00752-w

**Published:** 2021-01-06

**Authors:** Adeline Su Lyn Ng, Juan Wang, Kwun Kei Ng, Joanna Su Xian Chong, Xing Qian, Joseph Kai Wei Lim, Yi Jayne Tan, Alisa Cui Wen Yong, Russell Jude Chander, Shahul Hameed, Simon Kang Seng Ting, Nagaendran Kandiah, Juan Helen Zhou

**Affiliations:** 1 Department of Neurology, National Neuroscience Institute, Tan Tock Seng Hospital, Singapore, Singapore; 2 Neuroscience and Behavioral Disorders Program, Duke-NUS Medical School, Singapore, Singapore; 3 Centre for Sleep and Cognition, Department of Medicine, Yong Loo Lin School of Medicine, National University of Singapore, Singapore, Singapore; 4 Department of Neurology, National Neuroscience Institute, Singapore General Hospital, Singapore, Singapore; 5 Centre for Translational Magnetic Resonance Research, Yong Loo Lin School of Medicine, National University of Singapore, Singapore, Singapore

**Keywords:** Alzheimer’s disease (AD), Behavioral variant frontotemporal dementia (bvFTD), Higher-order cognitive networks, Network distinctiveness, Network segregation and integration

## Abstract

**Background:**

Alzheimer’s disease (AD) and behavioral variant frontotemporal dementia (bvFTD) cause distinct atrophy and functional disruptions within two major intrinsic brain networks, namely the default network and the salience network, respectively. It remains unclear if inter-network relationships and whole-brain network topology are also altered and underpin cognitive and social–emotional functional deficits.

**Methods:**

In total, 111 participants (50 AD, 14 bvFTD, and 47 age- and gender-matched healthy controls) underwent resting-state functional magnetic resonance imaging (fMRI) and neuropsychological assessments. Functional connectivity was derived among 144 brain regions of interest. Graph theoretical analysis was applied to characterize network integration, segregation, and module distinctiveness (degree centrality, nodal efficiency, within-module degree, and participation coefficient) in AD, bvFTD, and healthy participants. Group differences in graph theoretical measures and empirically derived network community structures, as well as the associations between these indices and cognitive performance and neuropsychiatric symptoms, were subject to general linear models, with age, gender, education, motion, and scanner type controlled.

**Results:**

Our results suggested that AD had lower integration in the default and control networks, while bvFTD exhibited disrupted integration in the salience network. Interestingly, AD and bvFTD had the highest and lowest degree of integration in the thalamus, respectively. Such divergence in topological aberration was recapitulated in network segregation and module distinctiveness loss, with AD showing poorer modular structure between the default and control networks, and bvFTD having more fragmented modules in the salience network and subcortical regions. Importantly, aberrations in network topology were related to worse attention deficits and greater severity in neuropsychiatric symptoms across syndromes.

**Conclusions:**

Our findings underscore the reciprocal relationships between the default, control, and salience networks that may account for the cognitive decline and neuropsychiatric symptoms in dementia.

## Background

Alzheimer’s disease (AD) and frontotemporal dementia (FTD) remain two of the most common causes of dementia under the age of 65. AD classically presents with impairment in short-term memory and visuospatial ability, sometimes with atypical deficits in other domains in younger patients [81]. Frontotemporal dementia frequently presents with behavioral and personality change, known as behavioral variant FTD (bvFTD) [89]. AD features typical atrophy in the posterior hippocampal-cingulo-temporal-parietal default mode network (DN) [50, 94]. In contrast, bvFTD relates to atrophy in the frontoinsular-striatal salience/ventral attention network (SVAN) [94, 95]. Increasingly, diagnostic uncertainty in the early stages of disease when minimal atrophy is seen on structural neuroimaging [58] calls for more sensitive imaging phenotype. The network-based neurodegeneration hypothesis proposes that neurodegenerative diseases target large-scale neural networks and regional vulnerability varies along network topology [86, 94]. Hence, charting the convergence and divergence in the alterations of brain network-based metrics caused by these dementia subtypes could help improve imaging marker sensitivity for differential diagnosis and symptoms mapping [9, 66, 72, 118].

Brain functional connectivity (FC) derived from spontaneous blood oxygenation level-dependent (BOLD) signals [12] during resting-state (or task-free) functional magnetic resonance imaging (fMRI) has been proposed to be a network-sensitive imaging method [49]. Functionally connected brain regions show highly synchronous slow fluctuation in BOLD signals and form functionally specialized clusters known as intrinsic connectivity networks, including the DN and SVAN [100, 118]. Importantly, functional connectivity alterations are different between neuropsychiatric and non-pathological aging [22, 56, 74], as well as among different dementia subtypes paralleling their atrophy signatures [67]. In early landmark studies, compared to well-matched healthy participants, AD patients showed lower DN connectivity to posterior parieto-occipital regions with intensified SVAN connectivity, while bvFTD patients showed attenuated SVAN connectivity most notably in the frontoinsular-striatal region with enhanced DN connectivity [117]. Furthermore, disease “epicenter”-based FC modeling was able to predict regional atrophy severity in each of the five distinct neurodegenerative disorders [116]. Studies like these underscored the value of studying network-specific FC. However, multiple pathological mechanisms may co-exist [116], and these pathological changes may be associated with other FC properties in dementia. First, alterations in inter-network FC are also frequently reported [17]. In particular, the regulatory relationship between the SVAN and DN (“anti-correlations”) is believed to be critical for normal cognition and may be compromised in dementia [23, 75]. Second, FC aberration can appear in brain networks outside the two reciprocating networks, such as the frontoparietal network associated with cognitive control, attention, and working memory, as previously reported in bvFTD [41]. Third, structural and functional insults in subcortical regions have been reported and may be useful for tracking progression [6] and subtyping [18]. Until now, although a number of studies have attempted to incorporate these phenomena into existing disease models by targeting subsets of networks or one dementia subtype [42, 90], few has employed a whole-brain connectome-based, multiple-syndrome approach.

Graph theoretical analyses on whole-brain functional connectome are able to quantify complex brain network topology including integration, modularity, and efficiency using both nodal and global indices [19]. Community structure analysis characterizes how network nodes cluster into densely interconnected “communities” to support behavioral and cognitive functions [31]. It has garnered extensive use in investigating neuropsychiatric disorders [15, 108]. For instance, comparing patients with early-onset AD (EOAD) and bvFTD [43], EOAD showed more severe global functional network alterations, while bvFTD showed relatively preserved global functional brain architecture but more focal alterations in nodal FC. Still, existing studies did not consider inter-network connections, subcortical regions, and brain modular structure simultaneously and investigated how brain network topological features contribute to cognitive and behavioral symptoms between AD and bvFTD [34, 53]. Such studies are also severely under-representing the Asian cohort, where aging and dementia rates are among the worlds’ highest.

To address these gaps, we applied whole-brain connectome approach to a well-characterized Asian cohort. We hypothesized that AD and bvFTD would show divergent abnormalities in the topological organization of functional brain networks extending into subcortical and inter-network connections. Patients with bvFTD would have more disrupted modular structure in the SVAN while patients with AD would show greater loss of network modularity in the DN. Both disorders would have damaged subcortical modular structure. We also predicted that the SVAN and subcortical topological changes would relate to behavioral problems while the topological changes in the other higher-order cognitive networks such as DN and frontoparietal control network would be associated with cognitive impairment.

## Methods

### Participants

Between April 2013 and January 2018, 168 participants were recruited from the memory clinics at the National Neuroscience Institute, Singapore [103, 107, 114] to undergo clinical, neuropsychological, and neuroimaging assessments. Participants were evaluated by a panel of cognitive neurologists and trained psychologists and deemed during consensus meetings to have fulfilled clinical diagnostic criteria for AD [73] and bvFTD [89]. Despite relying on predominantly clinical diagnostic criteria, which could contribute to mismatch with etiology, past studies suggested that focusing on patients with the most typical clinical profile representative of each subtype would still yield remarkable insights that are largely consistent with the underlying etiologies [119]. Healthy control subjects were required to have a Mini-Mental State Examination (MMSE) [44] score of 26 or above and a Clinical Dementia Rating (CDR) score of 0. Exclusion criteria included the following: (i) a history of alcohol or drug abuse; (ii) a current or known history of major depression and/or other neuropsychiatric conditions such as psychosis; (iii) comorbid neurodegenerative disease such as Parkinson’s disease; (iv) significant cerebrovascular disease such as cerebral amyloid angiopathy and/or prior stroke; (v) presence of contraindications to MRI.

Out of 168 participants, we included 111 participants in the analyses (50 subjects with AD,^1^ 14 subjects with bvFTD, and 47 healthy control (HC) older adults, see Table 1) who passed quality control of both structural MRI and task-free fMRI data and fulfilled motion-scrubbing criteria (see “Image Acquisition and Preprocessing” section). The three groups were well matched in their demographics including age, gender, ethnicity, and handedness. This study was approved by the SingHealth Institutional Ethics Review Board and written informed consent was obtained from each participant prior to recruitment into the study.

**Table 1:** Subject demographics and clinical characteristics

	HC (***n*** = 47)	AD (***n*** = 50)	bvFTD (***n*** = 14)	***p*** value
**Age (years)**	55.47–76.00 (63.20 ± 5.00)	53.58–73.85 (65.45 ± 5.87)	55.44–74.28 (62.05 ± 5.47)	–
**Gender (M/F)**	22/25	23/27	3/11	–
**Ethnicity (C/M/I/E/O)**	45/0/2/0/0	41/4/2/1/2	11/2/0/1/0	–
**Education (years)**	13.47 (3.57)	9.04 (3.84) ^h^	8.79 (2.30) ^h^	<.001*
**Handedness (R/L/A)**	45/1/1	48/0/2	13/1/0	–
**CDR-global**	0 (0)	0.76 (0.41) ^h^	0.92 (0.47) ^h^	<.001*
**MoCA**	28.15 (1.59)	17.32 (6.47) ^h^	17.29 (6.66) ^h^	<.001*
**MMSE**	29.02 (1.15)	20.82 (5.66) ^h^	21.21 (6.72) ^h^	<.001*
**Episodic**	0.34 (0.61)	−3.80 (1.51) ^h^	−2.54 (1.58) ^ha^	<.001*
**Executive**	0.74 (0.34)	−1.48 (1.87) ^h^	−2.10 (2.23) ^h^	<.001*
**Attention/working memory**	0.38 (0.96)	−0.28 (1.03) ^h^	−0.63 (1.06) ^h^	<.001*
**Language**	0.53 (0.62)	−0.78 (1.46) ^h^	−1.65 (1.51) ^ha^	<.001*
**VisuoSpatial**	0.75 (0.79)	−0.83 (0.98) ^h^	−1.14 (0.89) ^h^	<.001*
**NPI score**	0.45 (1.34)	2.76 (2.80)	9.10 (7.90) ^ha^	<.001*

Age is presented as range (mean ± standard deviation), and other items are present as mean (standard deviation). Superscript letters indicate whether group mean was significantly different from HC (h) or AD (a) based on post hoc comparisons (*p* **<** 0.05) following one-way ANOVA *Significant at *p* < 0.05 after Bonferroni correction for multiple comparisons

*Abbreviations*: *HC* healthy controls, *AD* Alzheimer’s disease, *bvFTD* behavioral variant frontotemporal dementia, *M/F* male/female, *C/M/I/E/O* Chinese/Malay/Indian/Eurasian/Others, *R/L/A* right/left/ambidextrous, *CDR-global* global Clinical Dementia Rating, *MMSE* Mini-Mental State Examination, *MoCA* Montreal Cognitive Assessment, *NPI score* total neuropsychiatric inventory severity score

### Neuropsychological assessments

The neuropsychological battery was performed by trained psychologists and assessed global cognition as well as five cognitive domains, namely episodic memory, executive function, attention, language, and visuospatial abilities. Global cognition was assessed using the MMSE and Montreal Cognitive Assessment (MoCA) tool [78]. Episodic memory was assessed using the ADAS-Cog 10-word delayed recall [76]; executive function was assessed using the Frontal Assessment Battery (FAB) [37]; attention was assessed using the Digit Span forwards test [104]; language was assessed using the 30-word Boston Naming Test (BNT) [69]; and visuospatial ability was assessed using the Weschler Adult Intelligence Scale (WAIS) block design test [104]. Performance on the individual tests in each of the five cognitive domains was transformed into *z*-scores and defined as the domain-specific *z*-scores. Of the 111 participants, 16 individuals did not have complete cognitive performance scores. As such, for further association analysis between network topology and cognitive performance, participants with complete neuropsychological scores (37 AD, 13 bvFTD, and 45 HC) were included. Additionally, the Neuropsychiatric Inventory (NPI) [32] was used to measure neuropsychiatric and behavioral symptoms. Only patients with complete NPI total severity scores across all components (25 AD and 12 bvFTD) were included in brain-NPI association analyses.

### Image acquisition and processing

All structural and functional images were collected using either a 3 T Siemens Tim Trio system (*n* = 39) or a 3 T Prisma Fit System (Siemens, Erlangen, Germany) (*n* = 72) after scanner upgrade. For each participant, high-resolution T1-weighted structural MRI was acquired using magnetization-prepared rapid-acquisition gradient (192 continuous sagittal slices, TR/TE/TI = 2300/2.28/900 ms, flip angle = 9°, FOV = 256 × 240 mm^2^, matrix = 256 × 240, isotropic voxel size = 1.0 × 1.0 × 1.0 mm^3^, bandwidth = 240 Hz/pixel). An 8-min task-free resting-state fMRI (eyes-open with fixation) was also acquired using a single-shot EPI sequence (36 axial slices, TR/TE = 2000/30 ms, flip angle = 90°, FOV = 192 × 192 mm^2^, matrix = 64 × 64, isotropic voxel size = 3.0 × 3.0 × 3.0 mm^3^, bandwidth = 2112 Hz/pixel).

Both functional and structural images were preprocessed using a standard pipeline based on FMRIB Software Library (FSL) [60] and Analysis of Functional NeuroImages software (AFNI) [30] following our previous approach [25, 27, 79, 80]. Preprocessing for the structural images included (1) image noise reduction, (2) skull stripping using the Brain Extraction Tool (BET), (3) linear and nonlinear registration to the Montreal Neurological Institute (MNI) 152 standard space, and (4) segmentation of the brain into gray matter, white matter, and cerebrospinal fluid (CSF) compartments. Preprocessing for the functional images included (1) dropping the first 5 volumes, (2) slice time and motion correction, (3) time series despiking and grand mean scaling, (4) smoothing using a Gaussian filter of 6 mm full-width at half maximum (FWHM), (5) band-pass filtering between 0.009 and 0.1 Hz, (6) detrending (both linear and quadratic trends), (7) coregistration to participants’ T1 images and then to MNI152 standard template using linear (FLIRT) and nonlinear (FNIRT) transformations, and (8) regressing out confounds of motion (six parameters), white matter (WM), and cerebral spinal fluid (CSF) and global signal.

To minimize the effects of head motion, motion scrubbing was applied on task-free fMRI data by discarding volumes with frame displacement (FD) > 0.5 mm or variance of temporal derivative of time course over voxels (DVARS) > 0.5% [84]. After motion scrubbing, participants with sufficient data (at least 4 min) were kept for analyses.

### Graph theoretical analysis of functional brain networks

**Fig. 1:**
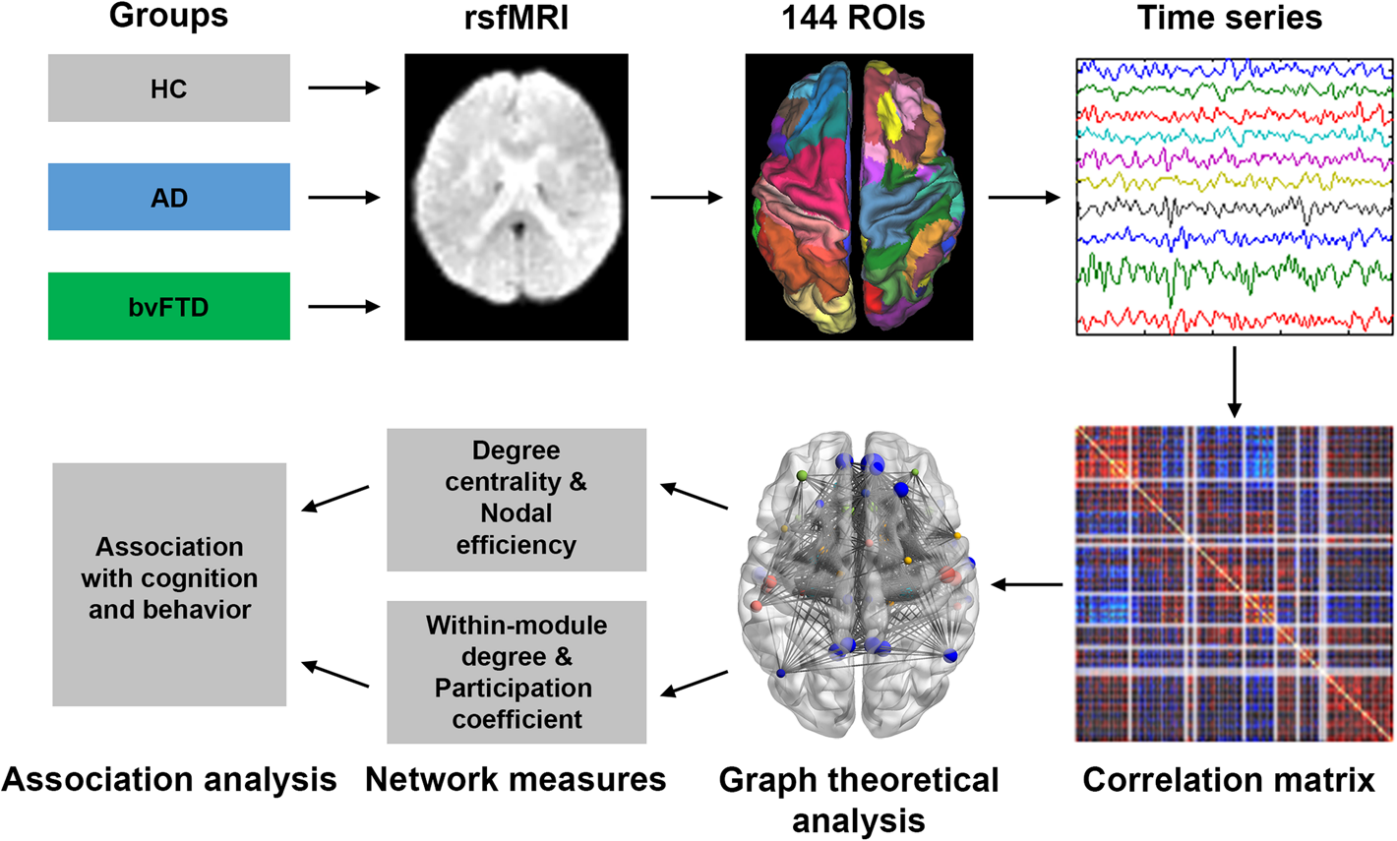
Study design schematic. We studied the network topological changes in patients with Alzheimer’s disease (AD) and behavioral variant frontotemporal dementia (bvFTD) compared to healthy controls (HC) and their association with cognition and behavior. Subject-level functional connectome (FC) was derived from task-free fMRI data based on pairwise Pearson’s correlations between the 141 regions of interests (see “Methods” for details). Graph theoretical nodal-wise metrics including degree centrality and nodal efficiency related to network integration as well as module participation coefficient and within-module degree related to network segregation and distinctiveness were computed for FC. Alterations in functional network topology metrics were compared across groups and subsequently associated with cognition and behavior

We constructed individual-level brain functional connectivity (FC) matrix from the preprocessed task-free fMRI data (Fig. 1). The mean fMRI time course was extracted from each of the 144 predefined regions of interest (ROIs) comprising 114 cortical regions [115] and 30 subcortical regions [105]. The cortical atlas was derived from clustering resting-state data from 1000 healthy young adults; each cortical ROI is affiliated with one of seventeen intrinsic functional brain networks that were highly replicable across individuals. Due to the limited brain coverage in certain scans, 141 ROIs (nodes) with full coverage in all scans were retained for network construction. We then calculated Pearson’s correlation between the time series of each pair of ROIs, resulting in a 141 × 141 weighted non-binarized FC matrix for each individual. All main diagonal elements and negative connections (edges) in the matrices were set to zero. Graph theoretical analysis was performed to compare the network topological properties among AD, bvFTD, and HC groups, focusing on their potential differences in brain network integration, segregation, and network distinctiveness (a concept closely related to segregation). We first thresholded the subject-level FC matrix using a range of costs (i.e., the ratio of the total number of edges to the maximal number of possible edges) from 0.09 to 0.4 in 0.01 steps. The cost range was determined based on the following criterion: (1) the small-worldness of the brain networks should be greater than 1 [68, 109, 111], (2) 80% of nodes in each network should be fully connected [8], and (3) the average number of connections per node should be larger than the log of the number of nodes [111]. We computed the following measures of network integration, segregation/distinctiveness, and modularity.

#### Integration

Network integration evaluates the ability of distributed brain regions to integrate and share information globally [91]. It was characterized by nodal degree centrality and efficiency in this study. These metrics were calculated at each cost threshold and then integrated across the entire range of cost thresholds to obtain a composite measure for each participant [48]. Degree centrality quantifies the capability of a node to catch whatever is flowing through the network. For a node *i*, it is defined as the sum of the weight of all edges that are directly linked to a node:

$$ {D}_i=\sum \limits_{j=1}^N{w}_{ij}, $$

where *w*_*ij*_ denotes the edges weight between node *i* and node *j*.

Nodal efficiency measures the ability of a node to propagate information with the other nodes in the network. For a node *i*, it is defined as:

$$ {E}_i=\frac{1}{\left(N-1\right)}\sum \limits_{i\ne j=1}^N\frac{1}{L_{ij}}, $$

where *L*_*ij*_ denotes the shortest path length between node *i* and *j*.

#### Segregation/module distinctiveness

Network segregation evaluates the ability of brain performing tasks in parallel, and segregated modules are useful for the development of specialized brain function [102]. We computed measures of within-module degree and participation coefficient to describe the segregation and distinctiveness of functional modules. The within-module degree is a measure of intra-module connectivity and evaluates the connectedness of a node to other nodes in the same module [52]. The participation coefficient, on the other hand, is a measure of inter-module connectivity and evaluates how evenly distributed connections of a node are across modules [52].

We first derived an empirical group-level community structure for each group using a two-stage consensus community detection proposed previously [10, 65]. In the first stage, community detection was conducted for each participant across a range of costs from 0.09 to 0.4 in 0.001 steps (311 thresholds in total). The community detection algorithm involved the following steps: (1) deriving a community structure for each of the 311 cost thresholds using the Louvain algorithm [13]; (2) constructing a co-classification matrix across all 311 community assignments in which each element represented the frequency that a given pair of brain nodes was assigned to the same community; and (3) performing a consensus-based clustering method [65] on the co-classification matrix to obtain a single final, consensus partition for each individual participant. In the second stage, we used the same steps to generate representative group-level community structures for each of the three groups, i.e., community detection was performed on a group-level allegiance matrix that was created by summing the consensus community assignments of all individuals in the group [16]. Community detection at both stages was performed using a range of γ parameters from 1 to 6 (resolution parameter *γ* determines the number of communities detected). We reported findings using individual-level *γ* = 3 and group-level *γ* = 2, as the brain network communities derived from the HC group with these two parameters best reflected the normative network organizations described in previous literature [24, 26, 115].

Based on the group-level modular structures, module-based network properties, which capture the role of nodes according to their pattern of intra- and inter-module connections [52], were analyzed. Specifically, we calculated the normalized within-module degree and participation coefficient at each node for each individual. The normalized within-module degree at each node *i* provides a measure of intra-modular connectivity and is defined as:

$$ {z}_i=\frac{k_{n_i}-{\overline{k}}_n}{\sigma_{k_n}} $$

where ${k}_{n_i}$ is the number of edges connecting the node *i* to other nodes in the module *n*, ${\overline{k}}_n$ is the average of ${k}_{n_i}$ over all nodes in the module *n*, and ${\sigma}_{k_n}$ is the standard deviation of the intra-modular degrees in the module *n*.

Participation coefficient at each node *i*, on the other hand, provides a measure of inter-modular connectivity and is defined as:

$$ {P}_i=1-\sum \limits_{n=1}^N{\left(\frac{k_{n_i}}{k_i}\right)}^2 $$

where *k*_*i*_ is the total degree of the node *i* in the entire network.

#### Modularity

Modularity refers to the degree to which modules/communities dissociate from each other and is a particularly important topological attribute for functional brain networks [46]. To examine the extent to which patient groups reorganized their network community structures relative to HC, we calculated the similarity of the group-level community partition between patient group and HC using the adjusted rand index [88] and a resampling method. We first resampled 20 subgroups from each group by randomly selecting 10 subjects with replacement in keeping with our focus on some representative group-level community structure based on the consensus assignments across individuals, instead of direct comparison of individual modularity structures. The group-level community structure for each of the 20 subgroups was then derived using the methods described above. We calculated the adjusted rand index between the community partition of each subgroup and the group-level modular structure obtained from all HC subjects at the whole-brain level, i.e., this all-HC structure served as a common reference template.

One step further, to evaluate the network-specific group differences in community structures, we calculated the adjusted rand indices of the community assignment within the combined default mode and executive control modules (DN-CN community), salience/ventral attention module (SVAN community), and subcortical module (subcortical community) between the two patient groups and the whole HC group separately. We chose these higher-order cognitive networks and subcortical regions based on the converging evidence of specific connectivity disruptions in AD and bvFTD [117, 119].

### Statistical analyses

#### Group differences in demographic and clinical characteristics

To examine group differences in demographic and clinical characteristics, we performed chi-square test on the categorical variables, and one-way analysis of variance (ANOVA) followed by post hoc two-tailed two-sample *t*-tests for pairwise comparisons on the continuous variables. Significance was set at *p* < 0.05 after Bonferroni correction.

#### Group differences in brain network topological metrics

To examine group differences in nodal degree centrality, nodal efficiency, within-module degree, and participation coefficient, we employed linear regression models on these nodal-wise metrics with group membership as the effect of interest while controlling for education years, scanner type, and number of frames after motion scrubbing. We reported statistically significant results at *p* ≤ .05 uncorrected for a complete picture of all potential differences between groups.

#### Group differences in modular structure

To compare the modular structure between groups, we performed one-way ANOVA followed by post hoc two-tailed two-sample *t*-tests on the adjusted rand indices separately for the whole brain, DN-CN community, SVAN community, and subcortical community. The Bonferroni correction was applied to correct for multiple comparisons at the level of *p* < 0.05.

#### Correlation of the nodal-wise network properties with cognitive performance and neuropsychiatric symptomatology

To assess the relationships between FC disruptions and cognitive performance as well as neuropsychiatric symptomatology, we correlated the nodal-wise network measures (nodal degree centrality, nodal efficiency, within-module degree, and participation coefficient) with the five cognitive domain scores as well as the total NPI severity scores across all patients. Only FC measures showing significant group differences were considered. Pearson’s correlation analysis was performed between FC metrics and cognitive/behavioral scores after regressing out age, gender, education years, and scanner type.

To further ascertain that the differences in graph theoretical measures between groups were more likely to be attributed to differences in topology instead of FC strength differences [106], we computed the mean FC (all edges surviving thresholding) for each participant at each cost, which was then aggregated across costs resulting in an area under the curve measure. This integrated FC was subject to the same linear model used in the main analyses. Furthermore, to account for potential confound by group differences in regional atrophy, we derived regional gray matter volume of each parcel (see Supplementary Methods) and included it and integrated FC as additional covariates in our analyses on the four nodal graph theoretical measures and correlation analysis with cognitional performance and NPI.

## Results

### Group differences in demographic and clinical characteristics

All three groups were well matched in demographics, such as age, gender, handedness, and ethnicity. The patient groups had lower level of education than HC. As expected, the two patient groups showed lower scores in CDR, MMSE/MoCA, specific cognitive domains, and neuropsychiatric symptoms (NPI) than NC. AD and bvFTD were comparable in education, CDR, MMSE/MoCA. AD displayed more severe episodic memory deficit than bvFTD (*t* = − 2.74, *p =* 0.008), while bvFTD displayed more severe language deficit (*t* = − 1.82, *p =* 0.07) and neuropsychiatric symptoms (*t* = 3.59, *p =* 0.001) than AD. No group difference was found between AD and bvFTD in terms of executive function, attention, and visuospatial ability. These profiles confirmed that our AD patients had a predominantly amnestic, multi-domain presentation.

### Group differences in brain network integration

**Fig. 2:**
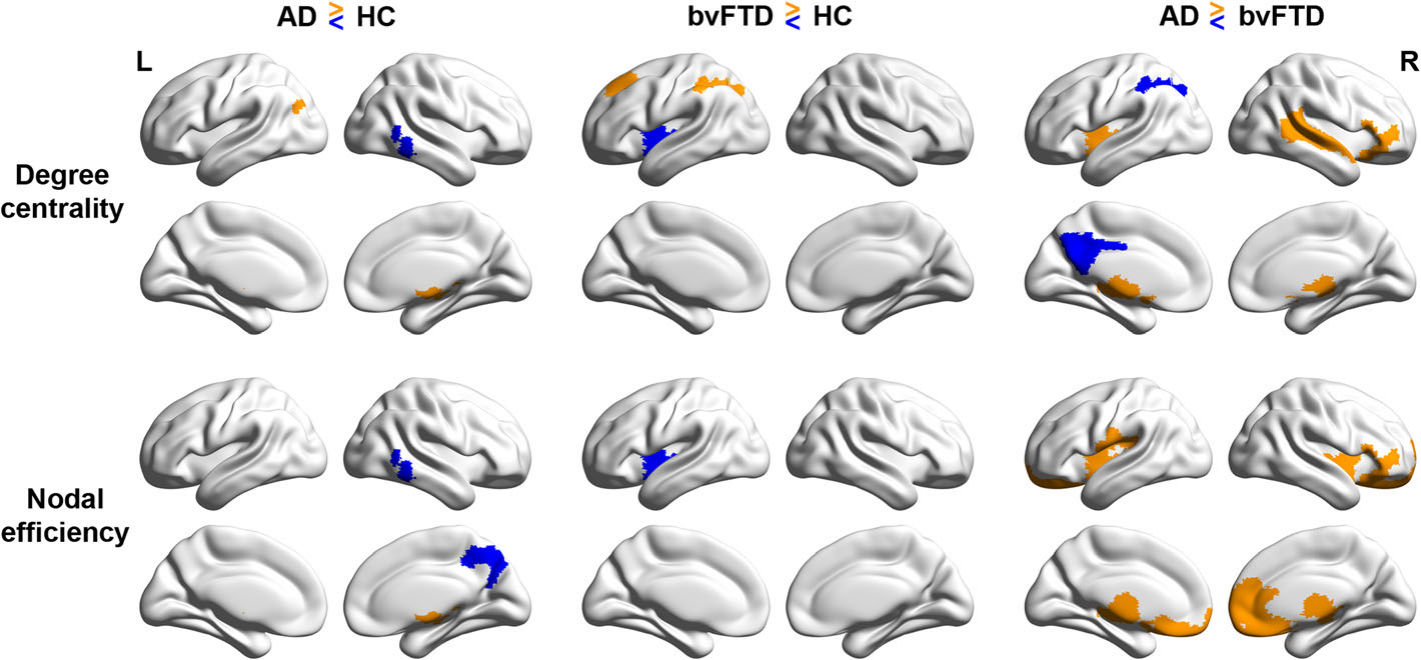
Network-specific alterations of degree centrality and nodal efficiency in AD and bvFTD individuals compared to controls. Brain surface plots indicating regions of increased (orange) or reduced (blue) degree centrality (top) and nodal efficiency (bottom) between groups. The results are displayed at the threshold of *p* < 0.05 (uncorrected). The findings of subcortical regions are further presented in Supplementary Tables 1 and 2

Groups did not differ in mean FC strength (*p* = .44) over the selected cost range (“integrated FC”), suggesting that the differences in network properties between groups could not be merely due to a group difference in connectivity strength [106]. The AD and bvFTD groups showed substantial divergent alterations in network integration in terms of degree centrality and nodal efficiency compared with HC group, especially in DN, CN, SVAN, and subcortical networks. Specifically, AD patients had lower degree centrality in the right temporal gyrus, but higher degree centrality in the thalamus and left inferior parietal lobule compared to HC (Fig. 2, top row). In comparison, bvFTD patients had lower degree centrality in the thalamus and insula of SVAN but higher degree centrality in the dorsal prefrontal cortex of DN and intraparietal sulcus of CN relative to HC. For nodal efficiency (Fig. 2, bottom row; Supplementary Tables 1 & 2), AD patients had lower nodal efficiency in the CN regions including right temporal gyrus and precuneus but higher nodal efficiency in thalamus compared to HC; bvFTD patients showed lower nodal efficiency in the insula and thalamus.

Similarly, direct contrasting between the two patient groups also revealed that compared to AD, bvFTD had lower degree centrality and nodal efficiency in the SVAN, limbic system, and subcortical regions including striatum and thalamus, but stronger degree centrality in posterior cingulate cortex and intraparietal sulcus, regions belonging to the DN and CN (Supplementary Table 1 & 2). Results remained largely unchanged after including integrated FC and regional atrophy (Supplementary Tables 1 & 2) as additional covariates.

### Group differences in brain network segregation and distinctiveness

We found that AD and bvFTD groups exhibited network-specific reductions in segregation and distinctiveness (i.e., lower within-module degree and higher participation coefficient) compared with HC. Such network topological changes were found mainly in the DN and CN for AD but SVAN and striatum in bvFTD.

**Fig. 3:**
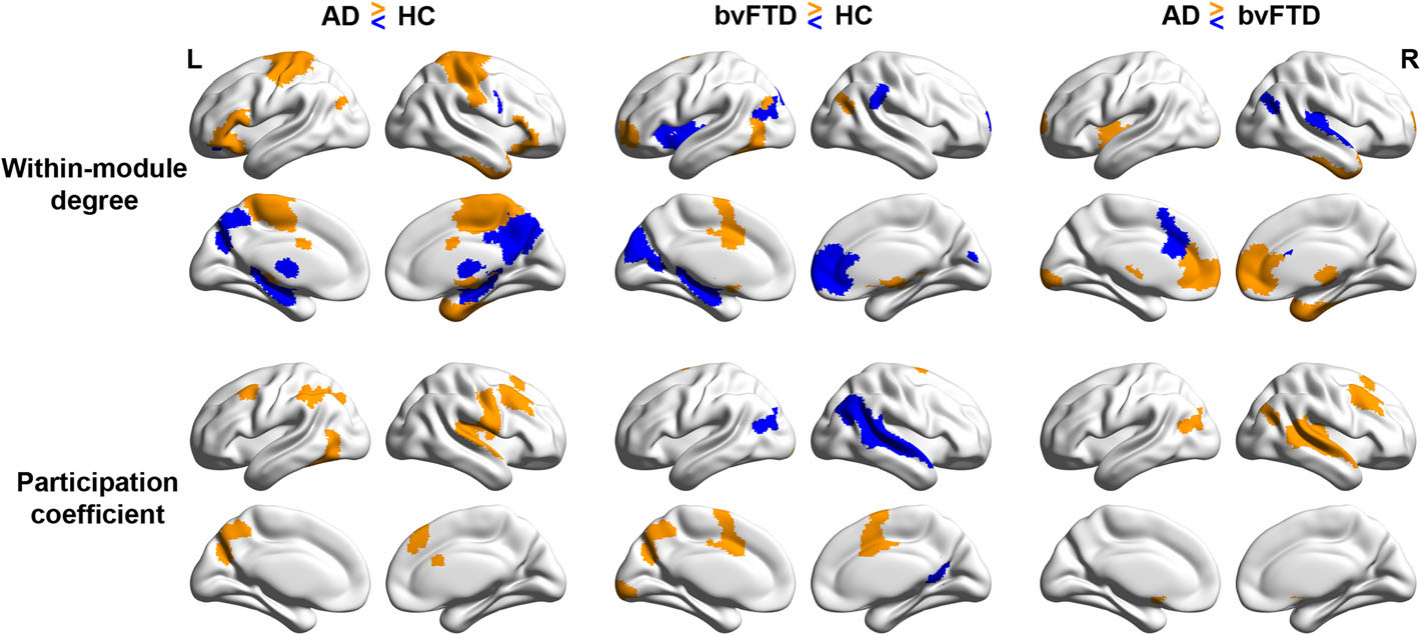
Network-specific within-module degree and participation coefficient changes in AD and bvFTD individuals. Brain surface plots indicating regions of increased (orange) or reduced (blue) within-module degree (top) and participation coefficient (bottom) between groups. The results are displayed at the threshold of *p* < 0.05 (uncorrected). The findings of subcortical regions are further presented in Supplementary Tables 3 and 4

Specifically, for within-module degree, a measure of intra-network connectedness, AD showed lower connectivity in the precuneus of the CN, posterior cingulate cortex of DN, and hippocampus but higher connectivity in the limbic system, medial parietal cortex of SVAN, the sensorimotor areas, and subcortical regions (Fig. 3, top left). In bvFTD, there was lower connectivity in the insular, prefrontal, and parietal ROIs of the SVAN and striatum but higher connectivity in the anterior cingulate cortex, prefrontal cortex, and inferior parietal cortex, regions belonging to the CN and DN (Fig. 3, top center). Interestingly, both higher and lower connectivity was observed in the subcortical regions. For participation coefficient, a measure of inter-network distinctiveness, compared to HC, AD showed higher coefficient (i.e., lower distinctiveness) in the CN (the inferior parietal cortex, prefrontal cortex, precuneus) and somatomotor cortex (Fig. 3 bottom left). In contrast, bvFTD showed lower network distinctiveness in the medial frontal cortex of SVAN, precuneus, visual cortex, and higher network distinctiveness in the parietal/occipital cortex and DN (including inferior parietal cortex and retrosplenial cortex) (Fig. 3 bottom center).

Comparing the two patient groups head-to-head, we found that AD patients had lower within-module degree in the inferior parietal regions and medial prefrontal cortex of the DN; bvFTD in contrast showed lower within-module degree in the limbic regions (temporal pole), the SVAN (insula and thalamus), and anterior/ventral DN regions (Fig. 3 top right, Supplementary Table 3). Similarly, AD showed lower network distinctiveness (i.e., higher participation coefficient) mainly in the DN and CN (including parietal/occipital cortex, inferior parietal cortex, dorsolateral prefrontal cortex) compared to bvFTD (Fig. 3 bottom right, Supplementary Table 4). Results remained largely unchanged after including integrated FC and regional atrophy (Supplementary Tables 3 & 4) as additional covariates.

### Divergent changes in brain functional modular organization in AD and bvFTD

**Fig. 4:**
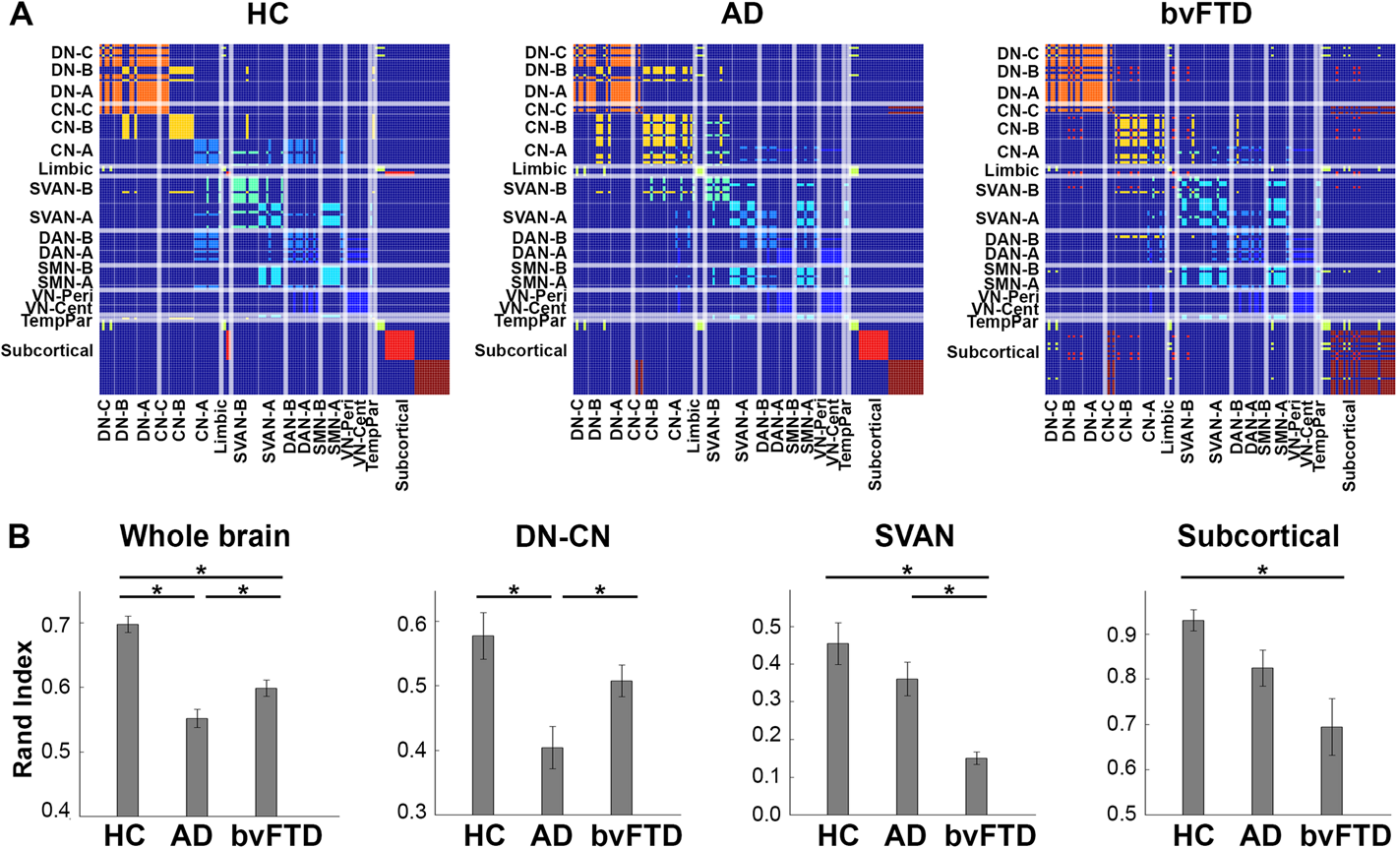
Differential fragmented network modular structure in patients with AD and bvFTD. **a** Consensus matrices showing community structures in HC, AD, and bvFTD groups. The edges connecting nodes in the same community are highlighted using the same color. **b** The similarity analysis of network community structures between patient groups and HC at the whole-brain network level and sub-network level (i.e., DN-CN, SVAN, and subcortical module). Error bars reflect standard error deviation of the mean adjusted rand index of each group relative to a referent community structure based on a resampling method (see text). For HC, they refer to the sampling variability of HC subgroups relative to the whole HC sample. Statistically significant group difference was indicated by * (*p* < 0.05, with Bonferroni correction for multiple comparison)

Analysis on brains’ modular organization not only revealed that patient groups showed global changes (overall network structure) compared with HC group, but there were also disease-specific alterations in the three modules of interest. Consistent with our hypotheses, the DN-CN module was disrupted in AD, and the SVAN and subcortical modules was disrupted in bvFTD. Overall brain network community structure differed between the three groups, as quantified by partition dissimilarity (F(2, 57) = 32.64, *p* < 0.001). The network community structure derived from sub-samples of both patient groups was significantly dissimilar, as evidenced by their lower rand indices compared to the HC sub-samples (AD: *t* = 7.73, *p* < 0.001; bvFTD: *t* = 5.57, *p* < 0.001). Of note, bvFTD showed the least similarity (bvFTD vs. AD, *t* = 2.52, *p* = 0.016), implying substantial network alteration in these patients (Fig. 4b). Further examination of community assignments for the patient groups revealed distinct disease-specific changes in our community patterns of interest (DN-CN: F(2, 57) = 7.68, *p* = 0.001; SVAN: F(2, 57) = 13.36, *p* < 0.001; subcortical: F(2, 57) = 7.04, *p* = 0.002). In the DN-CN modules, AD had a significantly different modular partition from HC (*t* = 3.57, *p* = 0.001) and bvFTD (*t* = 2.54, *p* = 0.015), reflecting lower segregation, or less distinctiveness, between DN and CN. In the SVAN module, bvFTD had a significantly different partition compared to HC (*t* = 5.21, *p* < 0.001) and AD (*t* = 4.35, *p* < 0.001). This was characterized by less segregation within the community (i.e., between SVAN_A and SVAN_B) as well as extension of the community to regions belonging to DN, CN, somatomotor, and subcortical networks in the reference HC structure. Finally, in the subcortical module, the partition of bvFTD was significantly different from HC (*t* = 3.57, *p* = 0.001), characterized by the integration between the striatum and thalamus in bvFTD. Subcortical community partition did not differ significantly between AD and bvFTD (Fig. 4b).

### Association of brain network topology with cognitive performance and neuropsychiatric symptoms

**Fig. 5:**
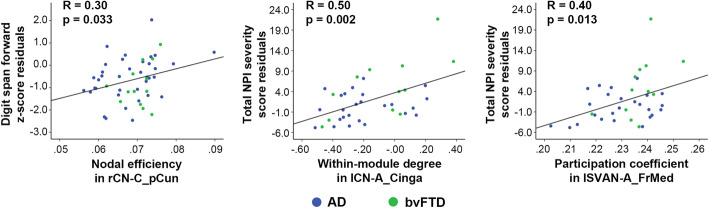
Functional network topology disruptions correlate with cognitive impairment and behavioral problems across patients with AD and bvFTD. Better performance in the digit span forward test was associated with higher nodal efficiency in the right precuneus of the executive control network (left, rCN-C_pCun). Neuropsychiatric symptoms evaluated by the total Neuropsychiatric Inventory (NPI) severity residual scores were associated with higher within-module degree in the left anterior cingulate cortex of the executive control network (middle, lCN-A_Cinga) and higher participation coefficient in the medial frontal cortex of the salience/ventral attention network (lSVAN-A_FrMed) across all patients

Among the graph theoretical measures that showed significant group differences between AD and bvFTD, lower nodal efficiency in the right precuneus of the CN was associated with lower attention performance (digit span forward *z*-score) (Fig. 5 left). In parallel, higher within-module degree in the left anterior cingulate cortex of the CN (Fig. 5 middle) and higher participation coefficient in the left medial prefrontal cortex of the SVAN (Fig. 5 right) across all patients were associated with more severe neuropsychiatric symptoms as measured by the total NPI severity scores. Results remained largely unchanged after including integrated FC and regional atrophy as additional covariates (Rs = .31, .49, and .39, respectively, all *p*s < .05).

## Discussion

To our knowledge, this is the first study providing direct evidence on the divergent impact of AD and bvFTD on whole-brain functional connectome and modular structure by incorporating inter-network and subcortical connections, paralleling the opposing patterns in atrophy and symptoms of the two disorders. Using graph theoretical measures, we found that both groups demonstrated distinct functional network topological alterations in network integration and segregation/distinctiveness, complementing earlier findings on the opposite changes in FC strength between the two syndromes [53] by offering a different level of network description. In line with the reciprocal network proposal [117, 119], both AD and bvFTD showed divergent breakdown of modular structure in networks known to be specifically affected in the respective syndrome: AD patients showed greater vulnerability in the DN and CN while bvFTD patients showed greater disruptions in the SVAN and subcortical regions. This study provides novel insights into how decline in network integration, segregation, and distinctiveness encompassing both cortical and subcortical regions contribute to cognitive and behavioral symptoms in neurodegenerative disorders.

### Divergent alterations in network integration, segregation, and modular structure in AD and bvFTD

Brain networks consistently reported with reduced functional connectivity (FC) in AD and bvFTD are those with the most neuropathologic changes and best associated with the symptom-deficit profiles [3, 4, 43, 61]. bvFTD patients consistently showed lower SVAN connectivity relative to AD [41, 117], while early-onset and late-onset AD patients showed lower DN connectivity relative to bvFTD [21, 117]. These findings parallel the characteristic memory-executive dysfunction in AD [71] and social–emotional dysfunction in bvFTD [92]. Nevertheless, these reduced within-network FC are only one facet of the large-scale network structure alterations in neurodegenerative disorders. Increasing number of studies have shown that transitions between a dedicated but isolated information processing mode (i.e., segregation) and a multimodal but costly communication mode (i.e. integration) are essential for flexible and adaptive cognition and behavior [11, 77, 99]. Our findings suggested that the most common neurodegenerative disorders may feature divergent disruptions in brain functional network integration and segregation.

#### Integration

AD patients showed reduced network integration mainly in the DN that is classically affected by AD pathology [82]. In contrast, bvFTD patients showed lower network integration (degree centrality and nodal efficiency) in cortical and subcortical areas belonging to the SVAN (insular, thalamus and striatum). Head-to-head comparisons between the two disorders further confirmed that the DN and SVAN may be altered antagonistically in these two dementia subtypes (in particular lower degree centrality and nodal efficiency in the SVAN but stronger degree centrality in posterior cingulate cortex and intraparietal sulcus contrasting bvFTD against AD), with AD and bvFTD attributable to divergent liberation and suppression of the two networks, as the reciprocal network model purports [119]. For instance, failed feedback from the DN (cf [28]) might explain the correlation between higher FC in SVAN and symptoms of hyperactivity (e.g., agitation, irritability, aberrant motor behavior, euphoria, and disinhibition) in AD [7].

We noted that the anterior and ventral medial prefrontal cortices (a/vmPFC), typically core regions of the anterior DN, evidenced higher nodal efficiency in AD compared to bvFTD. Past studies have repeatedly demonstrated the presence of subsystems within the DN, with the posterior DN [61] and the subsystem involving the medial temporal lobe [85] argued to be most affected by AD pathologies relative to other subsystems. Our findings thus may reflect this role as “cascade epicenter” by the posterior DN in AD. While a lack of measure of biomarker status (amyloid and tau; see “Limitations and future directions”) prevented us from discussing the possibility of sample heterogeneity, the higher efficiency in the anterior/ventral DN in AD than bvFTD could reflect compensatory neural activity [20, 110]. Interestingly, a/vmPFC is essential for socio-affective processing such as emotional regulation [39]; its higher efficiency in AD, together with altered SVAN activity [7], might account for increased emotional contagion often reported in these patients [119].

#### Modularity

While a few fMRI studies have compared network alterations between AD and bvFTD directly [43, 53, 117], no study has yet proceeded to directly compare their network community structure, a succinct system-level descriptor of brain network structure [108, 110]. Applying graph theoretical analysis and a resampling method to quantify module (dis)similarity, we found that both AD and bvFTD groups showed altered whole-brain community structure compared to HC. The patient groups showed significantly different community components from the HC both at the whole-brain and specific community levels (Fig. 4). Normalized rand index also suggested that the two patient groups differed in their community structures. Of note, this module dissimilarity between AD and bvFTD again demonstrated reciprocity at specific communities: the similarity with the referent default-control community was the lowest in AD, while the similarity with the referent salience/ventral attention community was the lowest in bvFTD.

#### Segregation

The divergent network alterations in the two subtypes were partly recapitulated in the community-derived nodal measures (as opposed to community-independent FC indices of degree centrality and efficiency), particularly derived to characterize network segregation. Compared to HC (Fig. 3 left column), AD patients showed lower within-module degree, a measure of connectedness inside the community, in the hippocampus, precuneus, and parietal cortices (intraparietal cortex and inferior parietal lobule), predominantly regions in the DN-CN community [51], while bvFTD patients showed lower degree in many SVAN and striatum regions [33, 95, 117]. Direct contrast between AD and bvFTD further showed that bvFTD had lower degree in the insula than AD, consistent the reciprocal model. These differences imply a compromised nodal importance within its own network.

Nevertheless, perhaps contrary to our hypothesis, this comparison also highlighted some curious differences, namely *higher* degree in the cingulum regions in bvFTD and *higher* degree in the anterior/ventral mPFC in AD. While a speculative compensatory explanation, as discussed above, may also apply here, the interpretation of the direction of the differences might be complicated by the fact that the patient groups did not share the same network structure. For instance, the DN subsystem B was detached from the referent (HC) DN module in AD but not bvFTD, while the two SVAN subsystems were less “distinctive” in bvFTD but not AD. Another interesting possibility is the presence of further “subtyping” within each syndrome. For example, further study may benefit from considering a dysexecutive variant of AD with predominant executive dysfunction symptoms [81].

In addition, compared to bvFTD, AD patients showed higher participation coefficient, a measure of inter-network connectedness, in many referent CN regions, suggestive of a loss of segregation between the DN and CN in AD. Loss of DN and CN distinctiveness may reflect declining network functional specialization or processing efficiency commonly observed in normal aging [24, 26], which might be accelerated by AD risk factors such as the possession of APOE e4 gene [80]. Together, these module-based findings complement our nodal FC results to highlight the putative reciprocal relationship between the DN and SVAN when these networks are compromised by dementia pathologies. Applying graph theoretical measures on resting-state EEG data, de Hann and colleagues [35] concluded that compared to controls with subjective memory complaints, the brain functional organization of AD was more random, while that of FTD was more (excessively) regular, possibly attributable to distinct pathologies. It would be of interest for future studies to examine if the specific modularity changes reported here explain the opposite topological deviations from “small-worldness” that typically characterizes optimal networks, amidst methodological variations between the two studies (e.g., definition of control group and FTD diagnosis).

### More severe subcortical network topological damage in bvFTD

Subcortical brain regions have been known to show selective, early vulnerability to frontotemporal lobar degeneration (FTLD) pathology [93] resulting in functional network alterations in bvFTD [41, 53, 87, 117]. Here, we found that subcortical connectivity was disrupted especially in bvFTD. Compared to AD and HC, bvFTD showed most community dissimilarity in the SVAN module and subcortical module, as a result of less segregated SVAN subsystems, “intrusion” of subcortical regions into cortical communities, and the remarkable integration of the striatum and thalamus regions. Interestingly, AD patients have higher integration in thalamus than bvFTD patients. Thalamic abnormality has been argued to be crucial in early AD for its close circuitry with other parts of the episodic memory system (the Papez circuit, [2]) and across the FTD spectrum for its putative key role in regulating multiple cognitive, motor, and socio-affective functions (e.g., atrophy [14];). In parallel, within-module degree and participation coefficient in many striatum ROIs were altered (both higher and lower) in bvFTD when compared to HC (degree) and AD (participation coefficient). Compared to controls, patients with bvFTD have previously shown reduced functional connectivity in frontoinsular and basal ganglia networks [43]; volumetric changes in striatal and thalamic regions were also reported in bvFTD [59] and other FTD syndromes (thalamus [14];). The cortico-striatal-thalamic connections are essential to many fundamental cognitive processes such as cognitive control [54], emotion regulation [64, 83], and reward-decision making [40]. Their FC alterations and modular modifications are consistent with deficits in socio-affective, autonomic processing, and executive function in bvFTD [38, 89].

To our knowledge, this is the first study to show alterations of subcortical structures in both nodal *and* modular organization in bvFTD. While more replications are needed to ascertain the direction and functional implications of the FC and organizational changes in the striatum and thalamus, finding from Jakabek and colleagues [59] suggested that these relationships might be dependent on regional structural connectivity. Specifically, their bvFTD patients had overall lower subcortical gray matter volume than HC, but striatal and thalamic regions with white matter connection to the medial prefrontal cortex, unexpectedly had significantly larger volume, possibly representing compensatory or maladaptive network remodeling. Such variations are likely to exert influences on their corresponding functional network profiles.

### Alterations in integration and segregation of associative networks correlated with cognition and neuropsychiatric symptom severity

Finally, we observed that brain regions showing group differences in network integration and segregation properties demonstrated reliable association with cognitive deficits and symptom severity across subtypes, suggesting that while the two subtypes have reciprocal clinical phenotypes and neural alterations, some deficits vary quantitatively along a “dementia spectrum” instead of showing stark qualitative differences, some of which might be better captured in between-network properties [17, 119]. Across all patients, better performance on attention was associated with higher integration (nodal efficiency) in the right precuneus of the CN. While designated as the control network regions, precuneus has been argued to be dissociable from the core dorsal DN and form its own posterior memory network (PMN) reflecting attentional and mnemonic processes such as novelty and familiarity of the to-be-remembered items [47], memory [96], and its retrieval [55]. Alterations of the intra- and inter-network PMN FC was shown to differentiate between normal and pathological (AD) aging [57, 63]. The linear positive association with an attentional test with a putative memory component [62, 112] is consistent with its putative functionality and further showcased its purported sensitivity to disease status in dementia in general. With more bvFTD patients included, future studies can investigate if the precuneus or PMN, considered separately of the DN, has differential diagnostic values (e.g., by examining interactive effect between diagnosis and network measures on outcomes).

Furthermore, worse NPI severity (higher score) was associated with lower modularity (higher participation coefficient) in the left frontomedial cortices of the SVAN across all AD and bvFTD patients. As discussed above, SVAN seemed to evidence the most loss of distinctiveness between subsystems in our bvFTD group compared to the rest. Consistently, more cross-talk between the frontomedial cortices of the SVAN (dorsal anterior cingulate cortex, dACC) with other non-SVAN regions, as indicated by higher participation coefficient, may indicate excessive and disruptive inter-network communication. Notably, we also found that higher NPI severity scores were associated with *higher* within-module degree in the left cingulate cortex of the CN. In the context of cognitive control, the dACC is responsible for mediating ongoing behavioral adaptation by providing a continuously updated prediction of expected cognitive demand to optimize future behavioral responses [36, 98]. While higher degree of the CN typically indicates better network integrity and predicts better cognition and disease symptoms [29], both AD and bvFTD evidenced a less segregated CN community structure compared to HC (Fig. 4, yellow). Similar to our perspective on the SVAN, but now *within* a module, higher connectedness between a node and its topological neighbors in an aberrant network may indicate loss of network distinctiveness rather than the more beneficial network integration. Since the two ROIs are very close to each other, we speculate that these organizational changes in the vicinity of the dACC together may reflect a unified inward-outward breakdown of the SVAN and CN in the two dementia subtypes that might be maladaptive than compensatory in nature, leading to behavioral symptoms.

### Limitations and future directions

This study has a few limitations. First, our sample size of bvFTD patients was relatively smaller compared to the AD group, although their clinical and demographical characteristics were well matched. Second, our results might be impacted by the choice of brain parcellation and its spatial resolution. Although consistent results have been found across parcellations [1, 97], one-scheme-fits-all sometimes risks oversimplification [5, 113]. Future work should focus on using individualized parcellations [25, 27] to derive and compare brain network topology. Third, while the group differences in nodal-wise graph theoretical measures did not survive multiple comparison correction, our key findings on the functional network organization (community structures) was not affected by this correction. Related, while our patient groups were comparable on overall FC strength, it will be very informative to systematically dissociate the contribution of FC strength and true topological changes to the observed group differences in the brain network properties [106]. Forth, our participants were grouped according to clinical diagnosis; while we adopted a high standard in our diagnosis and demonstrated their divergent clinical profiles, uncertainty such as mislabelling (e.g., due to clinical phenotype and pathology mismatch, [101]) may still be inevitable. Future studies with disease-specific biomarkers such as amyloid, tau, and cerebrovascular status will be important to studying disease heterogeneity [24–27]. Finally, our study focused mainly on the differences between the two dementia subtypes; conversely, studying their overlapping symptoms and the underlying neural substrates would be highly informative, an approach that has gained momentum in many neuropsychiatric conditions (e.g., [45, 70]).

In conclusion, we reported novel disease-specific disruptions in intrinsic functional network topology and modularity in AD and bvFTD, in particular highlighting the divergence patterns of the two disorders in higher-order associative cortical networks (default, salience, and control) and subcortical regions, reinforcing the significance of network-based functional disorganization in modulating cognitive and neuropsychiatric symptoms. Future longitudinal studies are needed to assess the pathological, genetic, and environmental factors leading to the divergent changes in functional organization of these two neurodegenerative disorders, especially in the early stage, and the potential impact on network-based breakdown along the disease continuum.

## Supplementary Information

**Additional file 1: **Supplementary Table 1. *T* statistics (with standard errors) of brain regions with significant group differences in degree centrality (*p* < .05 uncorrected). Supplementary Table 2. *T* statistics (with standard errors) of brain regions with significant group differences in nodal efficiency (p < .05 uncorrected). Supplementary Table 3. *T* statistics (with standard errors) of brain regions with significant group differences in within-module degree (p < .05 uncorrected). Supplementary Table 4. *T* statistics (with standard errors) of brain regions with significant group differences in participation coefficient (p < .05 uncorrected).

## Data Availability

The data that support the findings of this study are available from the corresponding author upon reasonable request.

## Abbreviations

AD: Alzheimer’s disease
APOE: Apolipoprotein E
a/vmPFC: Anterior/ventromedial prefrontal cortex
bvFTD: Behavioral variant frontotemporal dementia
CDR: Clinical Dementia Rating
CN: Control network
CSF: Cerebrospinal fluid
dACC: Dorsal anterior cingulate cortex
DN: Default (mode) network
EOAD: With early-onset Alzheimer’s disease
FC: Functional connectivity
FTLD: Frontotemporal lobar degeneration
HC: Healthy control
MMSE: Mini-Mental State Examination
MoCA: Montreal Cognitive Assessment
MRI: Magnetic resonance imaging
NPI: Neuropsychiatric Inventory
PMN: Posterior memory network
SVAN: Salience/ventral attention network
WM: White matter

## Footnotes

1 While 2/50 AD cases also had prominent language deficits at presentation suggestive of logopenic aphasia, their episodic memory deficits remained the most significant and prominent
