# Supplementary material for: Distinct network topology in Alzheimer’s disease and behavioral variant frontotemporal dementia
Source: Alzheimers Res Ther. 2021 Jan 6;13:13. doi: 10.1186/s13195-020-00752-w (PMC7786961; doi:10.1186/s13195-020-00752-w)
Supplement: Supplementary file 1 — Additional file 1: Supplementary Table 1. T statistics (with standard errors) of brain regions with significant group differences in degree centrality (p < .05 uncorrected). Supplementary Table 2. T statistics (with standard errors) of brain regions with significant group differences in nodal efficiency (p < .05 uncorrected). Supplementary Table 3. T statistics (with standard errors) of brain regions with significant group differences in within-module degree (p < .05 uncorrected). Supplementary Table 4. T statistics (with standard errors) of brain regions with significant group differences in participation coefficient (p < .05 uncorrected). [file 13195_2020_752_MOESM1_ESM.docx]

**SUPPLEMENTARY MATERIALS**

**Supplementary Method**

*Voxel-based morphometry (VBM).* Gray matter volume (GMV) probability maps for each participant were obtained from the T1 structural images using the VBM8 toolbox (dbm.neuro.uni-jena.de/software/) in Statistical Parametric Mapping (SPM12) (<http://www.fil.ion.ucl.ac.uk/spm/)>. Key steps included (1) segmentation into gray matter, white matter, and CSF; (2) creation of a customized template using nonlinear diffeomorphic anatomical registration (DARTEL) registration of the affine-registered segmented images; (3) registration of reference GMV and white matter volume probability maps to the DARTEL template to standard MNI152 space; (4) nonlinear modulation of preprocessed images by multiplying voxels with the nonlinear component of the Jacobian determinant, correcting for individual brain sizes; and (5) Regional GMV/atrophy was derived as the mean across voxels covered by each parcel (binary masks) from the same parcellation scheme as the fMRI data.

**Supplementary Table 1. *T* statistics (with standard errors) of brain regions with significant group differences in degree centrality (p < .05 uncorrected)**

| **ROIs** | **T values of pairwise comparisons** | | |
| --- | --- | --- | --- |
|  | **AD vs. HC** | **bvFTD vs. HC** | **AD vs. bvFTD** |
| LH_SalVentAttnA_Ins | -  - | **-2.34 (0.023)**  **-2.93 (0.005)** | 2.30 (0.025)  2.40 (0.020) |
| LH_ContA_IPS | -  - | **2.57 (0.013)**  **2.45 (0.018)** | **-3.07 (0.003)**  **-3.01 (0.004)** |
| LH_DefaultA_PFCd | -  - | **2.22 (0.031)**  **2.24 (0.029)** | -  - |
| LH_DefaultA_PCC | -  - | -  - | **-2.45 (0.017)**  **-2.43 (0.018)** |
| LH_DefaultC_IPL | **2.46 (0.016)**  **3.09 (0.003)** | -  - | -  - |
| RH_DorsAttnB_TempOcc | **-2.41 (0.018)**  **-2.15 (0.034)** | -  - | -  - |
| RH_SalVentAttnB_PFClv | -  - | -  - | **2.02 (0.048)**  **2.20 (0.032)** |
| RH_ContA_Temp | -2.43 (0.017)  - | -  - | -  - |
| RH_DefaultB_PFCv | -  - | -  - | 2.13 (0.037)  - |
| RH_TempPar | -  - | -  - | 2.26 (0.028)  2.36 (0.022) |
| 7LH_striatum_5 | -  - | -  - | **2.98 (0.004)**  **2.58 (0.012)** |
| 7RH_striatum_5 | -  - | -  - | **2.06 (0.044)**  **2.05 (0.045)** |
| 7LH_thalamus_1 | -  - | -  - | 2.30 (0.025)  2.04 (0.046) |
| 7LH_thalamus_2 | -  - | -  - | **3.10 (0.003)**  **3.04 (0.004)** |
| 7LH_thalamus_4 | -  - | -  - | 2.12 (0.039)  2.07 (0.043) |
| 7LH_thalamus_5 | -  - | -  - | **2.67 (0.010)**  **2.50 (0.015)** |
| 7RH_thalamus_2 | -  - | -  - | 2.20 (0.032)  2.06 (0.044) |
| 7RH_thalamus_3 | -  - | -**2.54 (0.014)**  **-2.36 (0.022)** | **3.04 (0.004)**  **2.99 (0.004)** |
| 7RH_thalamus_4 | -  - | -  - | **2.91 (0.005)**  **2.86 (0.006)** |
| 7RH_thalamus_5 | **2.66 (0.009)**  **2.61 (0.011)** | -  - | -  - |
| LH_DorsAttnA_TempOcc | -  - | -  **2.23 (0.030)** | -  - |
| LH_thalamus_6 | -  - | -  - | -  2.04 (0.046) |
| RH_VisCent_Striate | -  - | -  - | **-**  **-2.08 (0.042)** |

Positive *t* values represent the former group is greater than the latter group. Data in the parentheses are *p* values. *T* values in the first line of each cell were the group comparison results with the covariates of education, scanner type, and number of frames after motion scrubbing, while *t* values in the second line were the results with the additional covariate of nodal gray matter volume. Bolded cells represent significant group differences after further inclusion of the integrated FC as an additional covariate. Abbreviations: AD, Alzheimer’s disease; bvFTD, behavioural variant frontotemporal dementia; HC, healthy controls; LH, left hemisphere; RH, right hemisphere; Default A/B/C, default mode network (either A, B or C refers to the subnetwork); Cont, executive control network; DorsAttn, dorsal attention network; SalVentAttn, salience ventral attention network; Ins, insular cortex; IPL, inferior parietal lobule; IPS, intraparietal sulcus; PCC, posterior cingulate cortex; PFCd, dorsal prefrontal cortex; PFClv, lateral ventral prefrontal cortex; PFCv, ventral prefrontal cortex; Temp, temporal cortex; TempPar, temporal parietal cortex; TempOcc, temporal/occipital cortex.

**Supplementary Table 2. *T* statistics (with standard errors) of brain regions with significant group differences in nodal efficiency (p < .05 uncorrected)**

| **ROIs** | **T values of pairwise comparisons** | | |
| --- | --- | --- | --- |
|  | **AD vs. HC** | **bvFTD vs. HC** | **AD vs. bvFTD** |
| LH_SomMotB_S2 | -  - | -  - | 2.04 (0.046)  - |
| LH_SalVentAttnA_Ins | -  - | **-2.26 (0.028)**  **-2.83 (0.006)** | 2.26 (0.028)  2.32 (0.024) |
| LH_Limbic_OFC | -  - | -  - | **2.47 (0.016)**  **2.49 (0.016)** |
| RH_DorsAttnB_TempOcc | **-2.31 (0.023)**  - | -  - | -  - |
| RH_ContA_Temp | -2.51 (0.014)  - | -  - | -  - |
| RH_ContC_pCun | **-2.82 (0.006)**  **-2.46 (0.016)** | -  - | -  - |
| RH_SalVentAttnA_Ins | -  - | -  - | 2.13 (0.037)  - |
| RH_SalVentAttnB_PFClv | -  - | -  - | **2.04 (0.046)**  **2.06 (0.044**) |
| RH_Limbic_OFC | -  - | -  - | 2.64 (0.011)  2.31 (0.025) |
| RH_DefaultA_PFCm | -  - | -  - | 2.15 (0.036)  - |
| RH_DefaultB_PFCv | -  - | -  - | **2.71 (0.009)**  **2.36 (0.022)** |
| 7LH_striatum_4 | -  - | -  - | 2.24 (0.029)  - |
| 7LH_striatum_5 | -  - | -  - | **3.12 (0.003)**  **2.79 (0.007)** |
| 7LH_striatum_6 | -  - | -  - | 2.15 (0.036)  2.04 (0.046) |
| 7LH_striatum_7 | -  - | -  - | 2.22 (0.030)  2.15 (0.036) |
| 7RH_striatum_5 | -  - | -  - | **2.82 (0.007)**  **2.70 (0.010)** |
| 7LH_thalamus_1 | -  - | -  - | 2.63 (0.011)  2.51 (0.015) |
| 7LH_thalamus_2 | -  - | -  - | **3.41 (0.001)**  **3.43 (0.001)** |
| 7LH_thalamus_4 | -  - | -  - | 2.21 (0.031)  2.21 (0.031) |
| 7LH_thalamus_5 | -  - | -  - | **3.05 (0.003)**  **3.07 (0.003)** |
| 7LH_thalamus_6 | -  - | -  - | 2.32 (0.024)  2.44 (0.018) |
| 7RH_thalamus_2 | -  - | -  - | 2.33 (0.023)  2.28 (0.027) |
| 7RH_thalamus_3 | -  - | **-2.38 (0.021)**  **-2.20 (0.032)** | **3.04 (0.004)**  **3.06 (0.003)** |
| 7RH_thalamus_4 | -  - | -  - | **2.70 (0.009)**  **2.70 (0.009)** |
| 7RH_thalamus_5 | **2.11 (0.038)**  **2.20 (0.030)** | -  - | -  - |
| 7RH_thalamus_6 | -  - | -  - | 2.12 (0.039)  2.19 (0.033) |
| 7RH_thalamus_7 | -  - | -  - | 2.08 (0.042)  2.15 (0.036) |
| LH_DefaultC_IPL | -  **2.36 (0.020)** | -  - | -  - |
| RH_SomMotB_Ins | -  - | -  -2.06 (0.044) | -  - |
| RH_SalVentAttnB_IPL | -  - | -  - | -  2.01 (0.049) |
| RH_TempPar | -  - | -  - | -  2.04 (0.046) |
| 7LH_thalamus_3 | -  - | -  - | -  2.09 (0.041) |

Positive *t* values represent the former group is greater than the latter group. Data in the parentheses are *p* values. *T* values in the first line of each cell were the group comparison results with the covariates of education, scanner type, and number of frames after motion scrubbing, while *t* values in the second line were the results with the additional covariate of nodal gray matter volume. Bolded cells represent significant group differences after further inclusion of the integrated FC as an additional covariate. Abbreviations: AD, Alzheimer’s disease; bvFTD, behavioural variant frontotemporal dementia; HC, healthy controls; LH, left hemisphere; RH, right hemisphere; Cont, executive control network; DorsAttn, dorsal attention network; Default, default mode network; SalVentAttn, salience ventral attention network; SomMot, somatomotor network; OFC, orbitofrontal cortex; pCun, precuneus; PFCm, medial prefrontal cortex; PFClv, lateral ventral prefrontal cortex; PFCv, ventral prefrontal cortex; Temp, temporal cortex; TempOcc, temporal/occipital cortex.

**Supplementary Table 3. *T* statistics (with standard errors) of brain regions with significant group differences in within-module degree (p < .05 uncorrected)**

| **ROIs** | **T values of pairwise comparisons** | | |
| --- | --- | --- | --- |
|  | **AD vs. HC** | **bvFTD vs. HC** | **AD vs. bvFTD** |
| LH_VisCent_Striate | -  - | -  - | **2.51 (0.015)**  **2.53 (0.014)** |
| LH_VisPeri_ExStrSup | -  - | -2.16 (0.035)  -2.11 (0.039) | -  - |
| LH_SomMotA | 2.11 (0.037)  - | -  - | -  - |
| LH_DorsAttnA_TempOcc | -  - | 2.45 (0.018)  - | -  - |
| LH_DorsAttnA_ParOcc | **-**  **-2.14 (0.035)** | **-2.58 (0.013)**  **-2.47 (0.017)** | -  - |
| LH_SalVentAttnA_Ins | -  - | **-2.98 (0.004)**  **-3.23 (0.002)** | **2.79 (0.007)**  **3.01 (0.004)** |
| LH_SalVentAttnA_FrMed | -  - | 2.17 (0.034)  - | -  - |
| LH_SalVentAttnB_PFCv | -  - | -2.13 (0.037)  -2.12 (0.039) | -  - |
| LH_SalVentAttnB_OFC | **-2.93 (0.004)**  **-2.37 (0.020)** | -  - | -  - |
| LH_SalVentAttnB_PFCmp | -  - | -  - | **-2.38 (0.021)**  **-2.33 (0.023)** |
| LH_ContA_Cinga | **3.52 (0.001)**  **3.47 (0.001)** | **4.03 (< 0.001)**  **4.10 (< 0.001)** | -  - |
| LH_ContB_PFClv | -  - | 2.10 (0.041)  - | -  - |
| LH_ContC_pCun | **-2.12 (0.037)**  **-2.11 (0.038)** | -  - | -  - |
| LH_DefaultA_PFCm | -  - | -  - | **2.45 (0.017)**  **2.12 (0.039)** |
| LH_DefaultB_PFCv | **2.42 (0.017)**  **2.20 (0.030)** | -  - | -  - |
| LH_DefaultC_IPL | **2.61 (0.010)**  **3.19 (0.002)** | **3.15 (0.003)**  **3.60 (< 0.001)** | -  - |
| RH_SomMotA | 2.29 (0.024)  2.05 (0.043) | -  - | -  - |
| RH_SomMotB_Aud | -  - | -  - | **-2.05 (0.045)**  **-2.19 (0.032)** |
| RH_DorsAttnB_PostC | 2.27 (0.025)  2.07 (0.041) | -  - | -  - |
| RH_DorsAttnB_PrCv | -2.13 (0.036)  -2.17 (0.033) | -  - | -  - |
| RH_SalVentAttnA_ParMed | 2.44 (0.017)  2.12 (0.037) | -  - | -  - |
| RH_SalVentAttnB_IPL | -  - | **-2.51 (0.015)**  **-2.36 (0.022**) | -  - |
| RH_Limbic_TempPole | 2.60 (0.011)  2.84 (0.006) | -  - | 2.27 (0.027)  2.25 (0.028) |
| RH_ContA_Cinga | 2.57 (0.012)  2.53 (0.013) | -  - | -  - |
| RH_ContC_pCun | **-2.55 (0.012)**  **-3.01 (0.003)** | -  - | -  - |
| RH_DefaultA_PCC | -2.2 (0.030)  - | -  - | -  - |
| RH_DefaultA_PFCm | -  - | -2.44 (0.018)  - | **2.79 (0.007)**  **-** |
| RH_DefaultB_PFCv | 2.18 (0.032)  - | -  - | -  - |
| RH_DefaultC_IPL | -  - | **3.02 (0.004)**  **2.87 (0.006)** | -**2.79 (0.007)**  **-2.67 (0.010)** |
| AAL_37_Hippocampus_L | -2.90 (0.005)  - | -2.91 (0.005)  -2.34 (0.023) | -  - |
| AAL_38_Hippocampus_R | -2.44 (0.016)  - | -  - | -  - |
| 7LH_striatum_2 | -  - | -2.13 (0.038)  - | -  - |
| 7LH_striatum_4 | -  - | -3.12 (0.003)  - | -  - |
| 7LH_striatum_5 | -  **2.00 (0.049**) | 2.15 (0.036)  2.22 (0.030) | -  - |
| 7LH_striatum_6 | -  - | -2.83 (0.006)  -2.28 (0.027) | -  - |
| 7RH_striatum_5 | -  - | **2.34 (0.023)**  - | -  - |
| 7LH_thalamus_2 | 2.34 (0.022)  - | -  - | -  - |
| 7RH_thalamus_4 | -  - | -  - | **2.13 (0.038)**  **2.16 (0.035)** |
| 7RH_thalamus_5 | **2.71 (0.008)**  **2.51 (0.014)** | **2.28 (0.027)**  **2.30 (0.025**) | -  - |
| 7RH_thalamus_7 | -2.64 (0.010)  - | -  - | -  - |
| RH_DefaultA_IPL | -  - | **-**  **2.01 (0.05)** | -  - |
| 7RH_striatum_6 | -  - | **-**  **2.01 (0.05)** | -  - |
| LH_ContB_PFCmp | -  - | -  - | -  -2.05 (0.045) |
| RH_DefaultC_PHC | -  - | -  - | **-**  **-2.02 (0.048)** |

Positive *t* values represent the former group is greater than the latter group. Data in the parentheses are *p* values. *T* values in the first line of each cell were the group comparison results with the covariates of education, scanner type, and number of frames after motion scrubbing, while *t* values in the second line were the results with the additional covariate of nodal gray matter volume. Bolded cells represent significant group differences after further inclusion of the integrated FC as an additional covariate. Abbreviations: AD, Alzheimer’s disease; bvFTD, behavioural variant frontotemporal dementia; HC, healthy controls; LH, left hemisphere; RH, right hemisphere; Cont, executive control network; DorsAttn, dorsal attention network; Default, default mode network; SalVentAttn, salience ventral attention network; SomMot, somatomotor network; VisCent, central visual network; VisPeri, peripheral visual network; Aud, auditory cortex; Cinga, anterior cingulate sulcus; ExStrSup, superior extrastriate cortex; FrMed, medial frontal cortex; Ins, insular cortex; IPL, inferior parietal lobule; OFC, orbitofrontal cortex; ParMed, medial parietal cortex; ParOcc, parietal/occipital cortex; PCC, posterior cingulate cortex; pCun, precuneus; PFCm, medial prefrontal cortex; PFCmp, medial posterior prefrontal cortex; PFClv, lateral ventral prefrontal cortex; PFCv, ventral prefrontal cortex; PostC, postcentral gyri; PrCv, precentral ventral frontal cortex; TempOcc, temporal/occipital cortex; TempPole, temporal pole.

**Supplementary Table 4. *T* statistics (with standard errors) of brain regions with significant group differences in participation coefficient (p < .05 uncorrected)**

| **ROIs** | **T values of pairwise comparisons** | | |
| --- | --- | --- | --- |
|  | **AD vs. HC** | **bvFTD vs. HC** | **AD vs. bvFTD** |
| LH_VisCent_Striate | -  - | 2.03 (0.048)  - | -  - |
| LH_DorsAttnA_TempOcc | 2.03 (0.045)  2.62 (0.010) | -  - | -  - |
| LH_DorsAttnA_ParOcc | -  - | -2.35 (0.023)  -2.35 (0.023) | **2.76 (0.008)**  **2.86 (0.006)** |
| LH_SalVentAttnA_FrMed | -  - | **2.58 (0.013)**  **2.23 (0.030)** | -  - |
| LH_ContA_IPS | 2.19 (0.031)  2.03 (0.045) | -  - | -  - |
| LH_ContB_IPL | **2.32 (0.022)**  **2.19 (0.031)** | -  - | -  - |
| LH_ContC_pCun | **2.31 (0.023)**  **2.42 (0.017)** | **2.07 (0.043)**  - | -  - |
| LH_DefaultB_PFCl | **2.40 (0.018)**  **2.80 (0.006)** | -  - | -  - |
| RH_SomMotB_Cent | **2.61 (0.011)**  **2.23 (0.028)** | -  - | -  - |
| RH_SomMotB_S2 | 2.31 (0.023)  - | -  - | -  - |
| RH_SomMotB_Aud | 2.30 (0.024)  - | -  - | 2.12 (0.038)  2.08 (0.042) |
| RH_SalVentAttnA_FrMed | -  - | 2.12 (0.038)  2.07 (0.044) | -  - |
| RH_SalVentAttnB_Cinga | 2.06 (0.043)  2.06 (0.043) | -  - | -  - |
| RH_ContB_PFCld | **3.15 (0.002)**  **2.93 (0.004)** | -  - | **2.19 (0.032)**  **2.11 (0.039)** |
| RH_ContB_PFCmp | **2.41 (0.018)**  **2.41 (0.018)** | -  - | -  - |
| RH_DefaultA_IPL | -  - | **-2.09 (0.041)**  **-2.10 (0.040)** | -  - |
| RH_DefaultC_IPL | -  - | **-2.34 (0.023)**  **-2.19 (0.033)** | 2.71 (0.009)  2.47 (0.016) |
| RH_DefaultC_Rsp | -  - | -**3.32 (0.002)**  **-3.30 (0.002)** | -  - |
| RH_TempPar | -  - | **-2.77 (0.008)**  **-2.30 (0.025)** | **2.03 (0.047)**  **2.02 (0.048)** |
| 7LH_striatum_5 | -  - | -  - | 2.15 (0.036)  2.44 (0.018) |
| 7RH_striatum_1 | -  - | -  - | -2.01 (0.049)  - |
| 7RH_striatum_5 | -  - | -  - | **2.36 (0.021)**  - |
| 7LH_thalamus_3 | -  - | 2.12 (0.038)  2.02 (0.048) | -  - |
| AAL_41_Amygdala_L | -  2.20 (0.030) | -  - | -  - |
| 7LH_thalamus_3 | **-**  **2.21 (0.030)** | -  - | -  - |
| 7LH_thalamus_4 | **-**  **2.30 (0.024)** | -  - | -  - |
| LH_DefaultA_PFCm | -  - | **-**  **-2.28 (0.027)** | -  - |
| RH_DefaultA_PFCm | -  - | **-**  **-2.67 (0.010)** | -  - |
| 7LH_thalamus_2 | -  - | -  - | **-**  **2.15 (0.036)** |

Positive *t* values represent the former group is greater than the latter group. Data in the parentheses are *p* values. *T* values in the first line of each cell were the group comparison results with the covariates of education, scanner type, and number of frames after motion scrubbing, while *t* values in the second line were the results with the additional covariate of nodal gray matter volume. Bolded cells represent significant group differences after further inclusion of the integrated FC as an additional covariate. Abbreviations: AD, Alzheimer’s disease; bvFTD, behavioural variant frontotemporal dementia; HC, healthy controls; LH, left hemisphere; RH, right hemisphere; Cont, executive control network; DorsAttn, dorsal attention network; Default, default mode network; SalVentAttn, salience ventral attention network; SomMot, somatomotor network; VisCent, central visual network; Aud, auditory cortex; Cent, precentral gyrus; Cinga, anterior cingulate sulcus; FrMed, medial frontal cortex; Ins, insular cortex; IPL, inferior parietal lobule; IPS, intraparietal sulcus; ParOcc, parietal/occipital cortex; pCun, precuneus; PFCmp, medial posterior prefrontal cortex; PFCl, lateral prefrontal cortex; PFCld, lateral dorsal prefrontal cortex; Rsp, retrosplenial cortex; TempOcc, temporal/occipital cortex; TempPar, temporal parietal cortex.
